# Circadian regulation of human cortical excitability

**DOI:** 10.1038/ncomms11828

**Published:** 2016-06-24

**Authors:** Julien Q. M. Ly, Giulia Gaggioni, Sarah L. Chellappa, Soterios Papachilleos, Alexandre Brzozowski, Chloé Borsu, Mario Rosanova, Simone Sarasso, Benita Middleton, André Luxen, Simon N. Archer, Christophe Phillips, Derk-Jan Dijk, Pierre Maquet, Marcello Massimini, Gilles Vandewalle

**Affiliations:** 1 GIGA-Research, Cyclotron Research Centre–In Vivo Imaging Unit, University of Liège, 8 allée du 6 août, B30, B-4000 Liège, Belgium; 2 Walloon excellence in life sciences and biotechnology (WELBIO), Avenue de l'Hôpital, 1B 4000 Liège, Belgium; 3 Department of Neurology, Centre Hospitalier Universitaire de Liège, Domaine Universitaire du Sart Tilman, Bâtiment B 35, B-4000 Liège, Belgium; 4 Department of Biomedical and Clinical Sciences "L.Sacco", Universit degli Studi di Milano, via G. B. Grassi 74, 20157 Milano, Italy; 5 Fondazione Europea Di Ricerca Biomedica, Ferb Onlus, 16, V. Stresa, 20125 Milano, Italy; 6 Surrey Sleep Research Centre, University of Surrey, Egerton Road, Guildford GU2 7XP, UK

## Abstract

Prolonged wakefulness alters cortical excitability, which is essential for proper brain function and cognition. However, besides prior wakefulness, brain function and cognition are also affected by circadian rhythmicity. Whether the regulation of cognition involves a circadian impact on cortical excitability is unknown. Here, we assessed cortical excitability from scalp electroencephalography (EEG) responses to transcranial magnetic stimulation in 22 participants during 29 h of wakefulness under constant conditions. Data reveal robust circadian dynamics of cortical excitability that are strongest in those individuals with highest endocrine markers of circadian amplitude. In addition, the time course of cortical excitability correlates with changes in EEG synchronization and cognitive performance. These results demonstrate that the crucial factor for cortical excitability, and basic brain function in general, is the balance between circadian rhythmicity and sleep need, rather than sleep homoeostasis alone. These findings have implications for clinical applications such as non-invasive brain stimulation in neurorehabilitation.

## Abstract (web summary)

Cognitive performance is impaired after prolonged wakefulness, yet the contribution of circadian rhythms for proper brain function remains unclear. Here the authors show that cortical excitability measured using TMS exhibits robust circadian dynamics which is correlated with cognitive performance.

## Graphical abstract

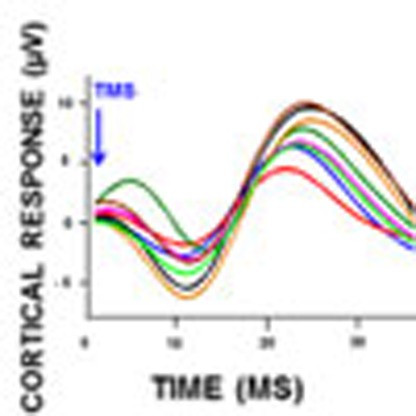

Wakefulness is associated with molecular, cellular and systemic changes in human brain function, which are deemed to negatively impact on cognition^1^,^2^. Deterioration of performance is, however, not a simple linear function of prior wakefulness duration. During the first ∼16 h of a normal waking day, human cognitive performance remains stable despite the concurrent build-up of sleep homoeostatic pressure. However, if wakefulness is extended into the biological night, cognitive performance deteriorates abruptly^3^,^4^. This reflects the influence of the circadian timing system, which counters the detrimental effect of sustained wakefulness during the day, until the end of the so-called evening ‘wake-maintenance zone' (WMZ)^5^,^6^. Subsequently, at night, the circadian system switches to a sleep promoting signal which favours sleep continuity, and opposes the progressive tendency to wake-up due to sleep pressure dissipation during sleep, up to the end of the early morning ‘sleep-promoting zone' (SPZ)^5^. Behavioural, neural and molecular correlates of the impact of the circadian timing system are being established^7^,^8^. However, its neuronal bases remain elusive^9^.

Cortical excitability, here defined as the strength of the response of cortical neurons to a given stimulation, reflects neuron reactivity and response specificity and is therefore a fundamental aspect of human brain function. It has been reported to increase with time awake in humans^10^. This may underlie performance decrements and greater seizure^11^ or hallucination^12^ propensity with sleep deprivation. Changes in human cortical excitability have been related to rodent data showing a linear increase with time awake in the firing rate and synchronization of cortical neurons^13^ and in the amplitude and slope of the local field potential evoked by electrical cortical stimulation^14^.

Synaptic function and structure have however also been reported to undergo marked circadian dependency^9^,^15^,^16^,^17^. Circadian variations in neuronal excitability have in fact been clearly established in invertebrates^18^. In humans, TMS-inferred corticospinal excitability (that is, TMS-evoked motor responses) was reported to depend on chronotype^19^ and to undergo a time-of-day influence, which appeared independent of sleep^20^. Sleep deprivation has been reported to have no effect^21^ or to decrease^22^ human corticospinal excitability, while it increased somatosensory cortex excitability^23^. It is therefore controversial, or it has at least not been conclusively established, whether, cortical excitability, similar to other aspects of human brain function, is modulated by both elapsed time awake and circadian phase.

Here we addressed this issue and investigated whether the circadian timing system impacts on human cortical excitability. We further investigated whether this potential circadian modulations of cortical excitability would correlate with the established circadian fluctuations in cortical synchrony across neuronal populations^14^,^24^ and behaviour^1^. We used transcranial magnetic stimulation coupled to high-density electroencephalography (TMS/EEG), as a non-invasive tool to gauge, *in vivo,* the time course of human cortical excitability during prolonged wakefulness. We hypothesized a circadian influence on cortical excitability to be most evident near the WMZ and SPZ and that individual variability in circadian signal strength as derived from endocrine markers (cortisol) to be related to the dynamics of cortical excitability. We further postulated cortical excitability to be associated with spontaneous waking EEG measures and performance assessments. Results confirm these hypotheses and reveal a robust circadian modulation of cortical excitability which correlates with changes in EEG synchronization and cognitive performance. The findings demonstrate that the balance between circadian rhythmicity and sleep need, rather than sleep homoeostasis alone, is crucial for cortical excitability regulation, and basic brain function in general.

## Results

**Figure 1:**
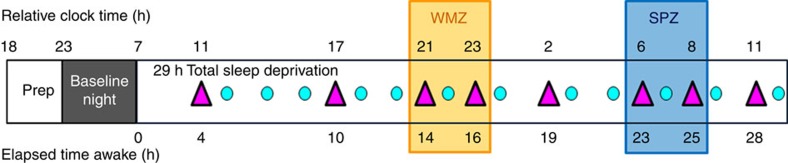
Experimental protocol. Participants underwent a 29 h sustained wakefulness protocol under constant routine conditions (no time-of-day information, constant dim light (<5 lux), external temperature and semi-recumbent posture, regular liquid isocaloric intake, sound proofed rooms). TMS-evoked EEG potential (TEP) were recorded eight times (>250 trials per session; violet triangles 
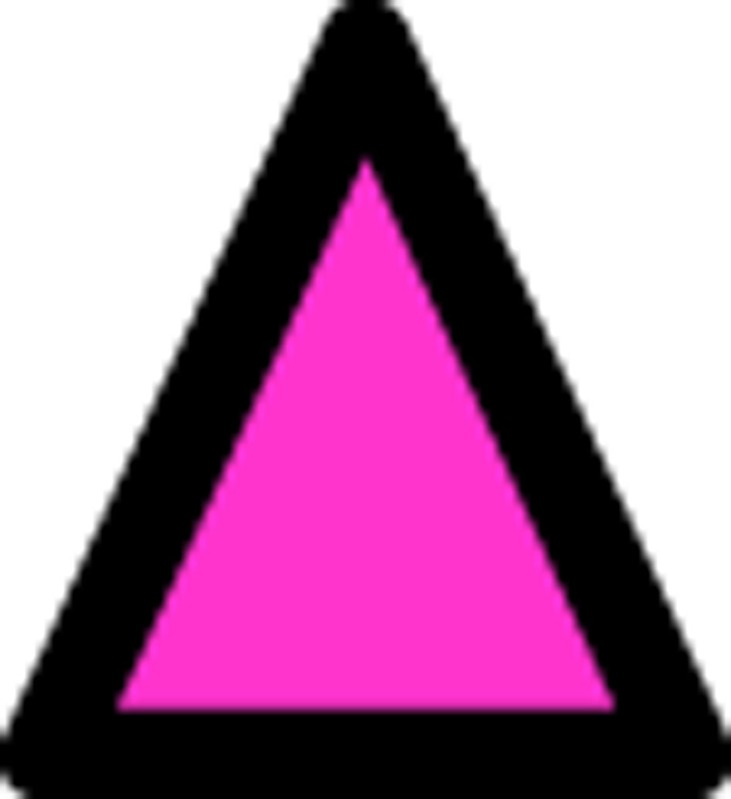
) and test batteries including the psychomotor vigilance task (PVT; turquoise circle 
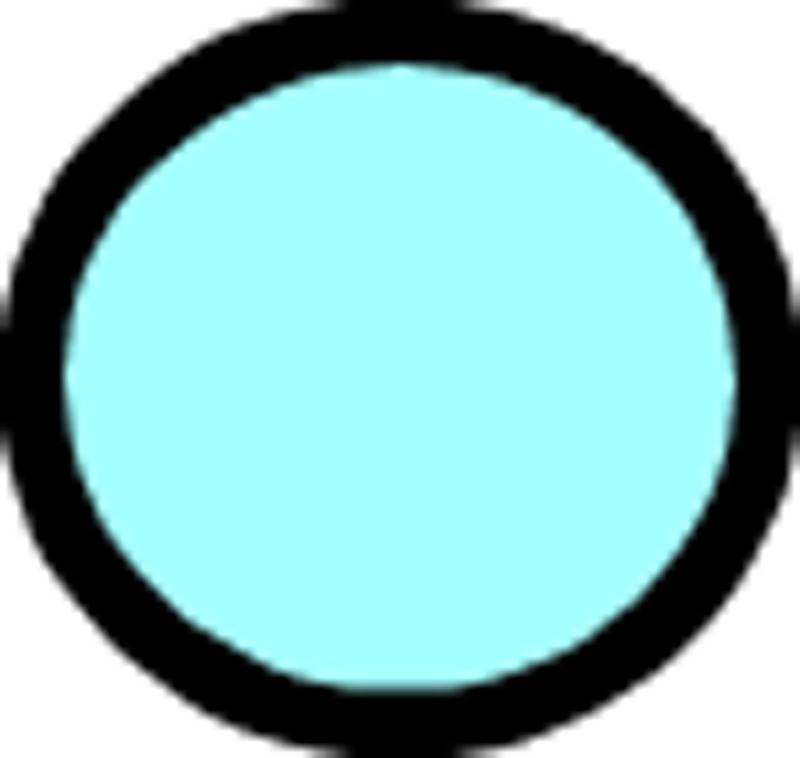
) were completed 12 times. TMS/EEG sessions were scheduled throughout the 29-h period with higher frequency around the wake-maintenance (WMZ) and sleep-promoting zones (SPZ), the timing of which was predicted based on habitual sleep times (data realigned a posteriori). During TMS/EEG sessions, participants performed a visual vigilance task consisting in maintaining a constantly moving cursor in the centre of a computer screen to assess simultaneous performance and exclude vigilance lapses. Saliva samples were collected hourly for melatonin and cortisol assays, together with subjective sleepiness and affect measures. Relative clock time displayed is for a participant with a 23:00–07:00 sleep–wake schedule. Prep: 5 preparatory hours, including test battery task practice (<5 lux). Baseline night: 8 h night of sleep in darkness at habitual sleep times and under EEG recording.

Following an 8-h nocturnal baseline sleep episode quantified by polysomnography, 22 healthy young men (22 years old±2.61; Supplementary Table 1 for participants characteristics), underwent eight TMS/EEG recordings during ∼29-h of continuous wakefulness. This sleep deprivation was conducted under strictly controlled behavioural and environmental conditions (constant routine protocol) to minimize external and internal factors masking circadian rhythmicity^25^ (Fig. 1). The frontal cortex supplementary motor area was chosen as stimulation target because it is highly sensitive to sleep deprivation^26^, as previously investigated using TMS/EEG^10^. TMS sessions were scheduled to adequately detect any predicted changes near the putative WMZ and SPZ. During TMS/EEG recordings, participants performed a simple visual vigilance task to assess performance as well as to exclude TMS/EEG segments during vigilance lapses from the analyses^10^. For the analyses all data were aligned to circadian phase as determined from individual melatonin profiles^27^. Participants were not provided with any information about time of day or the frequency and timing of assessments during the entire protocol to prevent any bias related to expectations on how one's brain state should change in relation to these variables (for example, it is 23:00, I must be sleepy).

Each TMS/EEG acquisition was preceded by a 2-min eyes open spontaneous waking EEG recording to extract theta frequency band power (4.5–7.5 Hz), an established marker of alertness and sleep need^24^. In between TMS/EEG sessions, participants also completed an auditory psychomotor vigilance task (PVT)^28^, used to monitor sustained attention. Subjective sleepiness and affect dimensions were assessed hourly. All these classical alertness-related measures exhibited typical and statistically significant variations during the protocol, with relatively stable values during the normal waking day period, followed by decrements during the biological night and subsequent partial recovery during the next day^3^ (PROC MIXED; *n*=22; *P*<0.002) (Supplementary Fig. 1).

### Non-linear cortical excitability change with wakefulness

**Figure 2:**
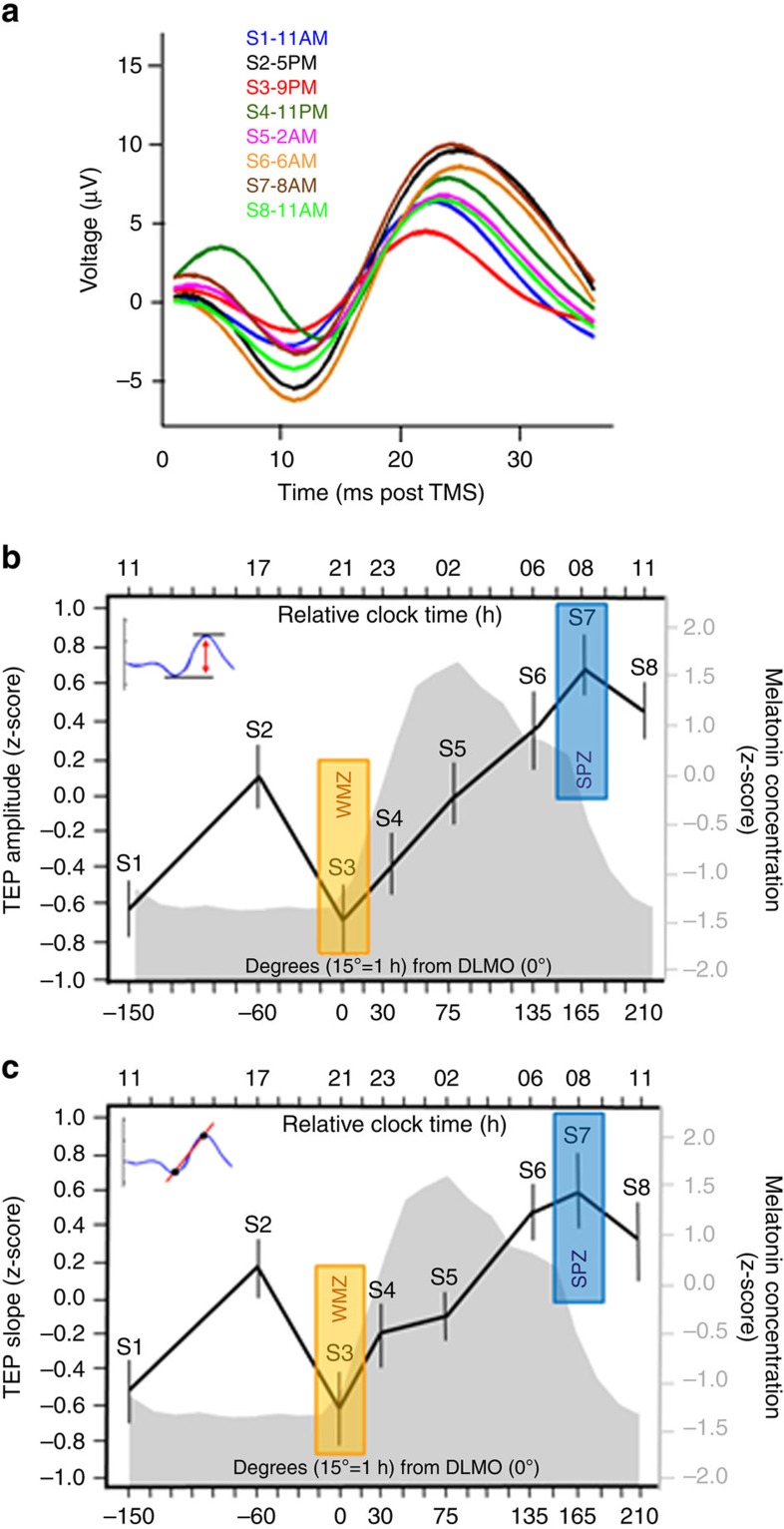
Non-linear changes in cortical excitability with wakefulness extension. (**a**) TMS-evoked potentials (TEP; 0–30 ms post TMS) measured at the electrode closest to the hotspot, averaged in each of the eight TMS/EEG sessions, in a representative participant (habitual sleep time: 23:00–07:00). Hotspot location was provided by the neuronavigation system. Time course of TEP amplitude (**b**) and slope (**c**) with respect to the circadian cycle. Data were averaged (mean±s.d.) after standardization (z-score) and realignment to individual circadian phase (*n*=22; melatonin secretion onset=0°). Mean z-scored melatonin profile is displayed in grey with respect to circadian phase (bottom *X* axis). The top *x* axis indicates relative clock time for a participant with a 23:00–07:00 sleep–wake schedule. Both TEP amplitude and slope significantly changed across the 29 h of sustained wakefulness (PROC MIXED; *n*=22; main effect of circadian phase: amplitude F_(7,128)_=8.17, *P*<0.0001; slope: F_(7,/129)_=5.91, *P*<0.0001). *Post hoc* analysis revealed (1) a significant increase from the first to the last session (*n*=22; S1 versus S8: amplitude: *P*_corr_=0.0025; slope: *P*_corr_ =0.0635], (2) a local decrease from the second afternoon session (S2) to the third evening session (S3) in the hypothetical WMZ (*n*=22; S2 versus S3: amplitude: *P*_corr_=0.037; slope: *P*_corr_=0.058), (3) a sharp increase during the biological night (*n*=22; S3 versus S7: amplitude and slope: *P*_corr_<0.0001], (4) ceasing after the seventh session, at the end of the theoretical SPZ (*n*=22; S7 versus S8: amplitude and slope: *P*_corr_>0.8).

**Figure 3:**
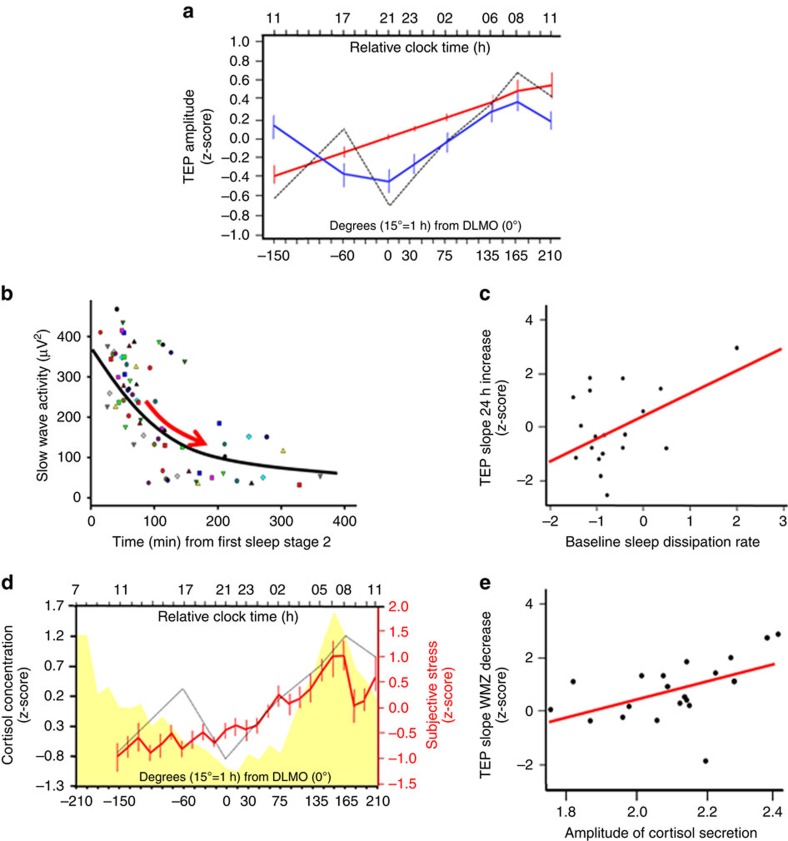
The circadian system modulates cortical excitability. (**a**) Individual cortical excitability measured by TEP amplitude (dashed line represents average z-scored TEP amplitude) was fitted with linear (**red**) and 24 h period sine-wave (**blue**) functions to mimic sleep pressure build-up and the circadian signal respectively. Error sum of squares (ESS) was <10 for both indices (amplitude: ESS linear fit=4.9, *P*<0.0001; ESS sine fit= 4.1, *P*<0.0001; slope: ESS linear fit=5.19, *P*<0.0001; ESS sine fit=4.24, *P*<0.0001). (**b**) Slow wave activity across the first four cycles of sleep baseline night was fitted to compute individual dissipation rate (schematically shown by **red arrow**). Each dot represents SWA of an individual sleep cycle (four identical symbols per participant). (**c**) Regression analysis showed that individual dissipation rate was positively correlated with the increase in cortical excitability from first to last session, recorded 24 h apart, at the same circadian phase, following a normal night of sleep and after sleep deprivation (*n*=18; amplitude: *P*=0.044; *r*^2^=.23; slope: *P*=0.036, *r*^2^=0.25). (**d**) Cortisol (**yellow**) and subjective stress (**red**) levels. Salivary cortisol concentration was not significantly different between the first and the last protocol samples, collected 24 h apart, at the same circadian phase, following a normal night of sleep and after sleep deprivation (*n*=22; F_(28,482)_=13.44; *P*<0.0001). Dashed line: shape of TEP z-scored amplitude dynamics. (**e**) Regression analysis revealed that individual fitted amplitude of cortisol secretion over the protocol was positively associated with the decrease in cortical excitability measured around the wake-maintenance zone (*n*=20; amplitude: *P*=0.017; *r*^2^=0.24; slope: *P*=0.023, *r*^2^=0.21).

Cortical excitability was inferred from the amplitude and slope of the first component of the TMS-evoked EEG potential (TEP; 0–30 ms post-TMS)^10^, measured at the electrode closest to the maximally stimulated brain location (hotspot). Both TEP amplitude and slope significantly changed with time awake (PROC MIXED; *n*=22; *P*<0.0001) (Fig. 2; see Supplementary Fig. 2 for non-standardized values). *Post hoc* analysis showed that cortical excitability increased globally from the first to the last session of the protocol (*n*=22; amplitude: *P*_corr_=0.025; slope: *P*_corr_=0.064). [All *post hoc* analyses for PROC MIXED were performed with Kenward-Roger's multiple comparison correction]. However, the dynamics of this increase was not linear. A marked significant local decrease was observed from the afternoon session (S2) to the evening session (S3), close to the onset of melatonin secretion, in the WMZ (amplitude: *P*_corr_=0.037; slope: *P*_corr_=0.058). Both amplitude and slope then significantly increased up to the seventh session (S7) around the maximum of cortisol secretion (Fig. 3d), at the end of the putative early morning SPZ (*n*=22; *P*_corr_<0.0001). This sharp increase appeared to subsequently cease 3 h later, in the last session (S8) of the protocol, which was no longer significantly different from the previous one (*n*=22; *P*_corr_>0.8).

Importantly, changes in estimated cortical excitability followed a similar pattern when inferring amplitude and slope of the TEP first component from a dipole computed at the hotspot, following EEG source reconstruction, that is, based on separate analyzes using signals from all available EEG electrodes (Supplementary Fig. 3).

These results confirm that human cortical excitability varies with extended wakefulness^10^, but reveal local non-linear variations compatible with a strong influence of the circadian timing system, in addition to a linear trend likely related to sleep homoeostasis.

### Cortical excitability correlates with circadian/sleep need markers

To further investigate this dual influence, we compared the predictive value of two different models to explain the observed time course of cortical excitability. The first fit consisted of a linear function representing the progressive build-up of sleep pressure^29^. The second fit comprised a 24 h period sine-wave function, centred on individual melatonin secretion onset, aimed at modelling the circadian signal^30^ (Fig. 3a). Both fits turned out to be good predictors of observed data, as indexed by low error indices.

In a next step, we related cortical excitability to independent individual standard measures of sleep homoeostasis and circadian rhythmicity. We first associated cortical excitability to a well-established marker of sleep pressure: NREM sleep slow wave activity (SWA)^2^. Individual dissipation rate of SWA reflects individual sleep homoeostasis efficacy in eliminating sleep pressure^31^. In our protocol, The first and last session were recorded 24 h apart at the same circadian phase (11:00 for an individual waking up at 07:00), such that their comparison should exclusively reflect the impact of time awake, that is, the build-up of sleep pressure. Regression analysis showed that SWA dissipation rate during the baseline night before sleep deprivation was positively associated with the build-up in cortical excitability in this interval (PROC REG; *n*=18; *r*^2^>0.22; *P*<0.037) (Fig. 3b,c).

Cortisol rhythm is characterized by declining values during the biological day, with a nadir near the WMZ, and rising values during the biological night with a peak at habitual wake time^32^. This is in contrast to the melatonin rhythm which is characterized by an on–off time course with very low levels during the day and high values during the night. We therefore evaluated a possible link between cortisol and cortical excitability dynamics. We found that cortisol levels covaried positively with increased TEP amplitude and slope over the entire protocol (Fig. 3d; analysis of covariance (ANCOVA); *n* =22; *r*^2^>0.24, *P*<0.0001). As it has been hypothesized that the amplitude of the cortisol rhythm may reflect the strength of a circadian signal^27^, we then investigated whether the amplitude of the cortisol rhythm is related to the non-linear change in cortical excitability. Regression analysis revealed a significant positive association between individual estimates of cortisol amplitude during the protocol and the decrease in cortical excitability from the afternoon session to the evening WMZ session (PROC REG; *n*=20; *r*^2^>0.21; *P*<0.023) (Fig. 3e).

Collectively, these findings speak to a critical role for sleep homeostasis on the dynamics of cortical excitability but they also indicate a relationship with the variation of a classical ‘hand-of-the clock' endocrine marker which putatively reflects individual circadian strength.

### Cortical excitability correlates with theta power and behaviour

**Figure 4:**
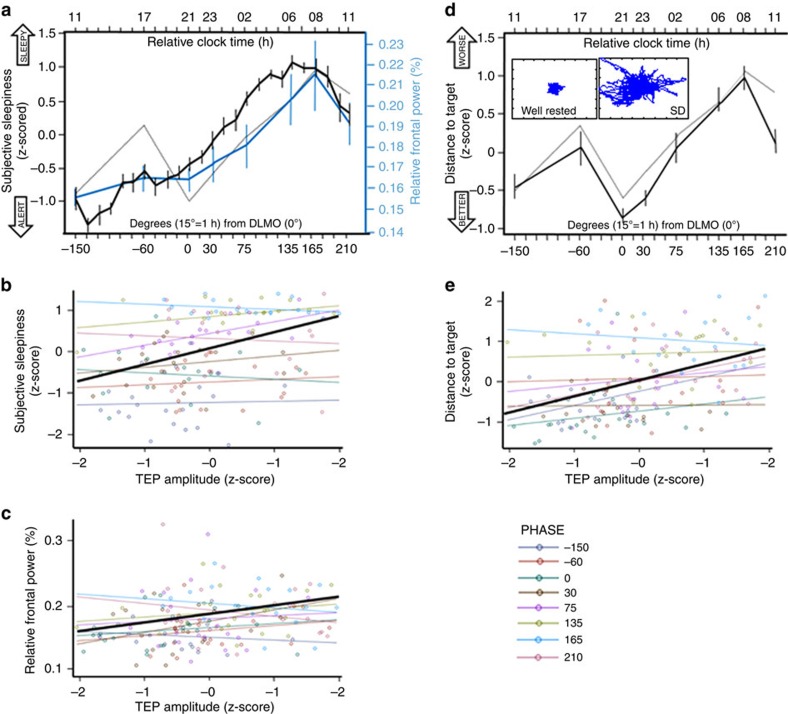
Cortical excitability dynamics is associated with changes in system-level brain function measures and in behaviour. (**a**) Time course of relative theta (4.5–7.5 Hz) power (%) in spontaneous waking EEG (**blue**) and subjective sleepiness (**black**) (mean±s.d.). Both variables showed significant variation over the sleep deprivation protocol (PROC MIXED; *n*=22; main effect of circadian phase: *P*<0.001; Supplementary Fig. 1 for details). Dashed line: shape of TEP *z*-scored amplitude dynamics. (**b**,**c**) ANCOVAs showed that relative theta power (**b**) (*n*=22; amplitude: *r*^2^=0.19, *P*=0.004) and subjective sleepiness (**c**) (*n*=22; amplitude: *r*^2^=0.69, *P*<0.0001) were significantly and positively associated with both indices of cortical excitability. Amplitude × circadian phase interactions was not significant (*P*>0.28). (**d**) Time course of performance to the vigilance task performed simultaneously to TMS/EEG recordings (mean±s.d.). The task consisted of maintaining a constantly moving cursor in the centre of a computer screen. Small inset depicts a representative well-rested and sleep-deprived (SD) session. Task performance (average distance kept from the screen center) significantly changed with time awake (PROC MIXED; *n*=22; main effect of circadian phase: F_(7,122)_=13.78; *P*<0.0001). (**e**) An ANCOVA revealed that vigilance task performance impairment was associated to TEP amplitude/slope increase (*n*=22; amplitude: *r*^2^=0.44, *P*<0.0001; slope: *r*^2^=0.43, *P*<0.0001). Amplitude/slope × circadian phase interaction was not significant (*P*>0.69).

Finally, we investigated whether the dynamics in cortical excitability, which arguably reflect a circadian influence, could constitute the neuronal bases for variations in individual brain system-level and behavioural measures, for which a circadian influence is widely accepted^1^,^3^. We found that TEP amplitude and slope significantly covaried with theta power over the frontal region across the 29 h of sustained wakefulness, with high cortical excitability associated with high theta power (ANCOVA; *n*=22; amplitude: *r*^2^=0.69, *P*<0.0001; slope: *r*^2^=0.69, *P*<0.0001) (Fig. 4a–c). This association was specific to theta power and was not observed for delta (0.75–4 Hz; ANCOVA; *n*=22; *r*^2^≤0.05; *P*≥0.95), alpha (8–12 Hz; ANCOVA; *n*=22; *r*^2^≤0.07; *P*≥0.77), sigma (12.5–18 Hz; ANCOVA; *n*=22; *r*^2^≤0.13; *P*≥0.13) and beta powers (18.5–30 Hz; ANCOVA; *n*=22; r^2^=0.08; *P*≥0.66) (Supplementary Fig. 4).

We then focused on the vigilance task which was performed simultaneously with the TMS/EEG recordings. Task performance showed non-linear changes across the protocol (PROC MIXED; *n*=22; main effect of circadian phase: F_(7,122)_=13.78; *P*<0.0001) and was significantly linked to cortical excitability dynamics such that higher indices of cortical excitability associated with worst performance (ANCOVA; *n*=22; *r*^2^=0.23, *P*<0.03) (Fig. 4d,e). Dynamics of cortical excitability also appeared to translate to the dynamics of subjective feelings. A last set of analyses showed that increases in subjective sleepiness (Fig. 4a,b) and negative affect (anxiety, stress and fatigue) and reductions in positive affect (mood, motivation and sociability) were related to increases in TEP amplitude and slope (ANCOVA; *n*=22; *r*^2^>0.4, *P*<0.0001). Altogether, these findings point towards a direct relationship between cortical excitability profiles and brain system-level or behavioural measure dynamics.

## Discussion

Our study confirms that cortical excitability, defined as the electrical reactivity of cortical neurons to a direct perturbation (TMS in the present case), is affected by the duration of wakefulness^2^,^9^,^10^, but it also demonstrates that cortical excitability is significantly modulated by circadian phase. An exclusive dependency on wakefulness duration would have led to a monotonic increase in cortical excitability with time awake. Our data show, however, that the initial increase in cortical excitability during a normal waking day returns to baseline value around the evening WMZ. In the context of our protocol, this evening excitability reduction can only be explained through an endogenous circadian influence independent of sleep, because the participants did not nap, had no direct knowledge of clock time and all environmental and behavioural conditions were kept constant. Reduction of cortical excitability would therefore represent a previously unappreciated marker of the circadian mechanisms by which performance is maintained at the end of a normal waking day, when sleep pressure is high.

Our results provide indeed a strong link between cortical reactivity, system-level measures of brain function (spontaneous waking EEG theta power) and behaviour (vigilance task, subjective feelings). Hence, the well-recognized non-linear variation in cognitive performance and subjective feeling during extended wakefulness^3^ appears to be related to basic aspect of neuronal function, that is, cortical excitability. During the biological night, cortical excitability exhibited a marked increase which coincided with decrements in performance, subjective feelings and objective EEG measure of alertness. Our data also suggest that the typical recovery observed in the morning of the second day of sustained wakefulness, as indexed by spontaneous waking EEG and behavioural measures, is mirrored by a decrease or at least a stabilization of cortical excitability. Further support for this statement would, however, require the demonstration of a significant reduction in cortical excitability following more extreme sleep deprivation.

Altogether, these findings strongly suggest that sleep is not the only process that regulates and restores neuronal function, as previously pointed out^9^. It has been suggested that mammals with weak circadian rhythms (for example, endotherm versus ectotherm) do not show evident circadian variations in synaptic function over the sleep–wake cycle^18^. This could explain in part why most previous studies have associated synaptic changes mostly with the sleep–wake rather than the circadian cycle^18^. Here we show that when vigilance state is kept constant, that is, participants remain awake in a constant routine protocol, circadian variations in neuronal and synaptic function become evident also in humans. The full separation and quantification of sleep homoeostasis and circadian influence is not possible using a constant routine protocol, during which wakefulness extension is always accompanied by concomitant changes in sleep pressure and circadian phase, and would require a forced desynchrony paradigm^5^. Our data show nevertheless that variations in cortical excitability are most obvious in individuals with strongest variations in spontaneous EEG activity, performance and subjective feeling as well as in those that have the largest amplitude in cortisol secretion, hypothesized to relate to the strength of the circadian wake promoting signal^27^. Cortical excitability also covaried with cortisol level which has been reported to rapidly affect synaptic function^33^,^34^. As a strongly circadian-driven signal, cortisol secretion could therefore mediate in part circadian variations in cortical excitability^18^,^35^. Cortisol co-variation with excitability could also reflect that they are both strongly influenced by the circadian system without a direct causal effect of cortisol. Core body temperature variation, also under circadian control, could equally contribute to the effect we report, as previously suggested^9^,^18^. However, the frequency specific effects of the circadian modulation of the wake EEG as assessed in a forced desynchrony protocol make it unlikely that all of the circadian effects can be attributed to temperature^24^.

In addition to its tonic circadian secretion, cortisol level also varies phasically with stress exposure. This phasic secretion has been suggested to mediate in part the effect of sleep deprivation in rodents^36^. We consider however that stress and stress-induced cortisol secretion are unlikely to have contributed significantly to cortical excitability dynamics in our protocol. First, subjective stress levels were relatively low in our sample, even though they did show previously reported significant circadian-related variations^37^ (*P*<0.0001) (Fig. 3d). Second, salivary cortisol levels of our participants did not exceed laboratory norms^38^. And finally, cortisol followed its typical circadian secretion profile^32^ in our sample, and cortisol levels at the end of the protocol were not significantly different from the beginning of the protocol, that is, at same circadian phase but 24 h apart (cf. Fig. 3; *P*_corr_=1).

Importantly, our results do not preclude a previously reported influence of sleep and sleep homoeostasis on synaptic function^10^. In our data, the overall build-up in cortical excitability, from the morning after a normal night of sleep to 24 h later following continuous wakefulness, is related to the individual differences in the dissipation of slow wave activity during sleep. This dissipation is mainly related to sleep homoeostasis, although for this variable, circadian influences are becoming evident^5^,^39^. Our findings supports a link between cortical excitability build-up during wakefulness and sleep-induced excitability reduction, at least when considering time points ∼24 h apart during extended wakefulness, that is, in the absence of a circadian confound.

Methodological differences are likely to explain the absence of circadian modulation of cortical excitability in previous studies^21^,^22^,^23^, including a study of ours^10^. In those studies time resolution was poorer (less assessments included over 24 h) and constant routine conditions were not implemented such that food intake, light exposure and physical activity for instance may have masked circadian rhythmicity^25^. In addition, in previous studies, the knowledge of time of day and of the number of assessments may have induced phasic motivation or engagement during experimental recordings^40^. Constant routine conditions, although strictly controlled should, however, not be considered as impoverished. Demanding test batteries are regularly performed, social interactions with researchers occur and participants engage in quiet activities between tests (reading, watching video, drawing, and so on—low light and acoustic levels). Therefore, we do not consider constant routine to have had a major impact on wake and use-dependent aspects of sleep homoeostasis, as participants' activities were intellectually demanding, resembled daily activity and included learning of novel information^2^. Finally, in prior studies, prior sleep–wake history was also not as carefully controlled as in the present study and data were not realigned to the onset of melatonin secretion, as a marker of circadian phase. This implies that in previous studies prior chronic sleep restriction may have not been fully dissipated before the experiment and that a 21:00 assessment in a given study^10^ could in fact represent a very different combination of sleep pressure and circadian phase than an assessment at 14 h of wakefulness in the present experiment, which also occurred at around 21:00 (for a participant waking up at 07:00).

The amplitude and particularly the slope of an EEG signal are considered to reflect neuronal synchrony and synaptic strength at the cortical level^14^. The variations in TMS-evoked EEG responses and their sharp overnight increase could therefore reflect a loss of discrimination or specificity of individual neurons and the impoverishment of firing repertoires of neuronal populations, which would jeopardize performance. Furthermore, global and local dynamics in neuronal synchrony have been demonstrated both during wakefulness and sleep^41^,^42^. As we stimulated a single brain area, we can only speculate about this global/local aspect. We delivered TMS over the frontal cortex because this region shows the most pronounced impact of sleep–wake history based on lower EEG frequency power variations^3^,^39^. The increase in these lower frequencies associated with wakefulness extension is global but also follows a fronto-occipital gradient^3^. This pleads for similar variations in cortical excitability over the entire brain that would be attenuated towards the occiput. Cortical excitability shows, however, region specific characteristics in the main frequency of a TMS-evoked EEG response in human^43^. Both gradual and maybe quite focal brain variations in the dynamics of cortical excitability are therefore likely and their extent deserves further investigation.

Modifications in cortical excitability imply changes in excitation/inhibition balance across subpopulations of neurons. This balance would therefore be under strong circadian influence, possibly through circadian changes in synaptic structure which is evident in many species other than humans^9^,^18^, through change in the extracellular milieu^44^, via a glial contribution, or through changes in the influence of brainstem and mesencephalic structure of the ascending arousal system^45^.

Cortical excitability increases have been associated with chronic insomnia^46^ and epilepsy^47^ and reductions have been observed in stroke^48^ and disorders of consciousness^49^. Combinations of increases and decreases have been reported in neurodegeneration^50^,^51^, depression^52^,^53^, possibly depending of the type and the stage of the disorder, as well as on time of day. Whether these abnormalities are sustained over the entire 24 h sleep–wake cycle or are only transient is unclear. Likewise, whether the dynamics of cortical excitability over the circadian cycle is altered in those pathological conditions is also not known.

Circadian disruption is, for instance, very common in Alzheimer disease and is deemed to contribute to cognitive impairment in those patients^54^. A time-of-day variation in the occurrence of seizures is also well established in certain forms of epilepsy^55^. Our data also imply that there may be optimal times of day for neurorehabilitation approaches which attempt to restore normal cortical activity in neurological conditions, either through cognitive intervention programs^56^ or non-invasive neurostimulation^57^. A circadian influence on cortical excitability may explain for instance why neurostimulation using TMS or transcranial electric stimulation (TES) fails to induce consistent improvement across clinical studies in Alzheimer's disease or stroke patients^57^,^58^. A full characterization of the temporal profile of cortical excitability in clinical populations may contribute to the development of TMS or TES neurorehabilitation strategies.

As a whole, our study, based on a relatively large sample and on repeated assessments over the 24 h day–night cycle, provides novel insights in the regulation of neuronal and synaptic function in healthy individuals and demonstrates that cortical excitability dynamics is strongly influenced by circadian rhythmicity. Its full characterization holds promise for cognitive enhancement in healthy and clinical brains^58^,^59^.

## Methods

### Participants

The study was approved by the Ethics Committee of the Medicine Faculty of the University of Liège. Participants gave their written informed consent after the nature and possible consequences of the studies were explained and received a financial compensation. Twenty-four healthy Caucasian men (18–30 years) were enroled. Women were excluded from the study as changes in ovarian hormones may influence cortical excitability in humans^60^. Other exclusion criteria included: (1) BMI ≤18 and ≥25; (2) psychiatric history, severe trauma, sleep disorders; (3) addiction, chronic medication; (4) smokers, excessive alcohol (>14 doses per week) or caffeine (>3 cups per day) consumption; (5) night shift workers during the last year; (6) transmeridian travel during the past 2 months; (7) anxiety or depression; (8) poor-sleep quality; (9) excessive self-reported daytime sleepiness. One participant was excluded due to a melatonin phase-delay >6 h compared with the remainder of the sample, and one due to low EEG recording quality. Thus, data presented here include 22 participants. Supplementary Table 1 summarizes the demographic characteristics of the final study sample. Participants were recruited based on a polymorphism in *PERIOD3* (*PER3* variable number of tandem repeat, with 4 or 5 repeats)^61^, but genotype was ignored in the analysis given the limited sample size of PER3^5/5^ genotype (7 *PER3*^*5/5*^for 15 *PER3*^*4/4*^).

### Experimental protocol

Participants first completed a ‘pretest' TMS/EEG session to determine the optimal TMS parameters providing artefact-free EEG recordings. The left or right supplementary motor area (SMA) was set as stimulation target for right or left handed, respectively. This brain area was identical to^10^ and was chosen for the following reasons: (1) similar to the entire frontal lobe, the SMA is exquisitely sensitive to sleep pressure, including at the neuronal level, as indicated in a previous EEG-TMS experiment^10^; (2) it plays a key role in cognitive performance, and is heavily connected to the prefrontal cortex^62^; (3) its stimulation does not trigger muscle activation, sources of EEG signal contamination.

The second step consisted of a laboratory polysomnographically monitored habituation night to exclude potential sleep disorders. During the 7 days preceding the study, participants kept a regular sleep–wake schedule of 8 h sleep duration (±15 min). Compliance was verified using wrist actigraphy (Actiwatch, Cambridge Neurotechnology, UK) and sleep diaries (Supplementary Table 1). Participants were requested to abstain from all caffeine- and alcohol-containing beverages and from intense physical activity for 3 days preceding the study.

For the experiment *per se*, participants arrived at the laboratory ∼6 h before their habitual sleep time. They were maintained in dim-light from there on (5<lux, except for sleep episode in complete darkness) and trained twice on the behavioural test battery. They then slept for an 8 h sleep baseline episode starting at their habitual bedtimes (Supplementary Table 2). The TMS-compatible electrode cap was placed upon awaking before the 29 h of sustained wakefulness period (sleep deprivation) under constant routine conditions (that is, light ca. 5 lux, temperature ca. 19 °C, regular isocaloric liquid meals and water, semi-recumbent position and no time-of-day information, sound proofed rooms) during which they did not interact with other participants but could engage conversation with research staff (outside test periods). These conditions aim to minimize external and internal factors masking circadian rhythmicity^25^.

Spontaneous quiet waking EEG and TMS-evoked EEG potentials (TEP) were recorded eight times during sleep deprivation to cover the entire near-24 h circadian cycle, with higher session frequency around the circadian WMZ and SPZ^5^ (11:00, 17:00, 21:00, 23:00, 02:00, 06:00, 08:00, 11:00, for a subject sleeping from 23:00 to 07:00; Fig. 1). Behavioural test batteries were carried out 12 times during the sleep deprivation period in between EEG sessions (12:00, 14:00, 16:00, 18:, 20:00, 22:00, 00:00, 03:00, 05:00, 07:00, 09:00, 00:00). Subjective sleepiness and affect dimensions were assessed hourly by the Karolinska Sleepiness Scale (KSS) and a Visual Analogical Scale (VAS), respectively. Saliva samples for melatonin and cortisol assays were also collected hourly.

### TMS-evoked EEG responses acquisition

TMS pulses were generated by a Focal Bipulse 8-Coil (Eximia; Nexstim, Helsinki, Finland). Stimulation target (SMA) was located on individual structural MRI by means of a neuronavigation system (Navigated Brain Stimulation; Nexstim). This device allows for reproducible evoked EEG responses^63^ and precise target location (FDA approval for presurgery). Each session included between 250 and 300 trials. Interstimulus interval was randomly jittered between 1,900 and 2,200 ms. Coil recharge was set at 900 ms post-TMS. Total number of stimulations of the eight EEG/TMS sessions was well below safety recommendations^64^.

TMS responses were recorded with a 60-channel TMS-compatible EEG amplifier (Eximia; Nexstim), equipped with a proprietary sample-and-hold circuit equipment guaranteeing TMS artifact-free data 8 ms post TMS^65^. Electrooculogram was recorded with two additional bipolar electrodes. Participants wore the EEG cap during the entire constant routine protocol and electrodes impedance was kept below 5 kΩ. Signal was band-pass-filtered between 0.1 and 500 Hz and sampled at 1,450 Hz. Each EEG/TMS session ended with a neuronavigated digitization of the location of each electrode.

Auditory EEG potentials (AEP) evoked by the TMS and bone conductance were minimized by diffusing a continuous loud white masking noise through earplugs and applying a thin foam layer between the EEG cap and the TMS coil, respectively^63^. Each session was followed by a ‘sham' session consisting in 30–40 TMS pulses delivered parallel to the scalp while white noise was diffused with the same level. Absence of AEP was checked online on Cz between 0 and 300 ms post TMS.

### Spontaneous waking and sleep EEG acquisition

Spontaneous quiet waking EEG was recorded prior to each TMS session using the same 60-channel TMS-compatible EEG (+2 EOG) amplifier (Eximia; Nexstim). Participants were instructed to fix a black dot during 2 min while relaxing and suppressing blinking.

Sleep EEG data were recorded using a V-Amp 16 amplifier (Brain Products GmbH, Gilching, Germany) according to 10/20 system). The habituation night montage consisted of a full PSG with five EEG channels (Fz, Cz, Pz, Oz, C3) referenced to left mastoid (A1), two bipolar electrooculogram (EOG), two bipolar electrocardiogram channels, two bipolar electrodes placed on a leg to check for periodic movements and an oximeter for sleep related breathing disorder detection. Baseline night montage consisted of 11 EEG channels (F3, Fz, F4, C3, Cz, C4, P3, Pz, P4, O1 and O2) referenced to left and right mastoids (A1 and A2), two bipolar EOG and two bipolar electromyogram (EMG) channels. EEG data were digitized at a sampling rate of 500 Hz.

### TMS vigilance task

While recording TMS-evoked EEG responses, participants performed a visual task (CTT) to monitor their vigilance level^10^. The task consisted of keeping a constantly randomly moving cursor on a target located in the centre of a computer screen, using a trackball device. Performance to the task was computed as the average distance, in pixels, between the cursor and the target during EEG/TMS recording (normalized according to the duration of the session). Transitory lapses of vigilance resulted in temporary increases of the target—cursor distance which could be automatically detected offline. A lapse was defined as a time when the cursor was located outside of a central 200 by 200 pixel box surrounding target following >500 ms from the last trackball movement. The lapse period included the period between the last trackball movement and the lapse detection. TMS-evoked responses occurring during and <1 s from a lapse were discarded from the analyses.

### Psychomotor vigilance task

Participants were required to press a computer space bar as soon as an auditory signal, presented at a random interval of 3–7 s, occurred. The PVT lasted 5 min. Session performance was inferred from the median reaction time following removal of lapses (>500 ms), anticipation (<100 ms) and error (>3,000 ms)^28^.

### Saliva collection for melatonin and cortisol assays

Saliva samples were first placed at 4 °C, prior centrifugation and congelation at −20 °C within 12 h. Salivary melatonin and cortisol were measured by radioimmunoassay (Stockgrand Ltd, Guildford, UK), as previously described^66^. Of a total of 624 samples, 546 were analysed in duplicate for melatonin concentration. The limit of detection of the assay for melatonin was 0.8±0.2 pg ml^−1^ using 500 μl volumes. Of a total of 631 samples, 631 were analysed in duplicate for cortisol concentration. The limit of detection of the assay for cortisol was 0.37±0.05 nmol l^−1^ using 500 μl volumes^67^.

### TMS/EEG data analysis

TMS/EEG data pre-processing was computed using Statistical Parametric Mapping 12 (SMP12, http://www.fil.ion.ucl.ac.uk/spm/) implemented in Matlab 2011a (The Mathworks Inc, Natick, MA). Continuous EEG recordings were successively re-referenced to the average of all good channels, low-pass filtered at 80 Hz, downsampled from 1,450 to 1,000 Hz, and high-pass filtered at 1 Hz, split into epochs between –101 and 300 ms around TMS pulses, and baseline corrected -101 to −1.5 ms pre-TMS periods. Robust averaging was applied to compute the mean evoked response of each session^68^.

Cortical excitability was inferred from the amplitude and slope of the first EEG component (0–30 ms) of the TEP measured at the artifact-free electrode closest from the hotspot (that is, brain location with highest TMS-induced electrical field estimated by the neuronavigation system). The latter electrode was always located in the stimulated brain hemisphere. It could vary across participants but remained constant at the individual level. TEP amplitude and slope were also extracted from a reconstructed signal at the hotspot using localization of equivalent current dipole.

### Spontaneous waking and sleep EEG analyses

Waking EEG data were analysed with MATLAB (2011a, The Mathworks Inc, Natick, MA). Data pre-processing was performed using Statistical Parametric Mapping 12 (SPM12, http://www.fil.ion.ucl.ac.uk/spm). Artefacted channels were rejected after visual inspection. Continuous EEG recordings were downsampled from 1,450 to 500 Hz. Data were then manually and visually scored offline for artefacts (eye blinks, body movements, and slow eye movements). Power spectral densities were computed using a fast Fourier transform on artifact-free 4-s, overlapping by 2 s, using the Welch's method (pwelch function in MATLAB 7.5.0). EEG activity was computed over frontal region (FP1, FPz, FP2, AF1, AFz, AF2, F7, F3, F1, Fz, F2, F4 and F8) for delta (0.75–4 Hz), theta (4.5–7.5 Hz), alpha (8–12 Hz), sigma (12.5–18 Hz) and beta (18.5–30 Hz) frequency bands over the entire 2-min recording.

Sleep EEG recordings were re-referenced to the average of both mastoids and band-pass filtered between 0.5 and 25 Hz. Data were visually inspected for artefact and manually scored for sleep stages on a 30-s epoch basis using FASST (an SPM compatible toolbox^69^), according to AASM criteria^65^. One baseline night was excluded from analyses because of poor quality of the recording (*n*=21). NREM-REM sleep cycles were determined according to Feinberg and Floyd. Power spectra were computed using a fast Fourier transform on successive 4-s epochs, overlapping by 2 s and weighted by a Hanning window.

### Statistics

All statistical analyses were performed with SAS version 9.3 (SAS Institute, Cary, NC, USA). For TEP amplitude and slope, cortisol level, KSS and PVT measures, standardization was provided by a *z*-score at individual level. TMS vigilance task was normalized by dividing performance to the duration of task and then *z*-scored. Frontal waking theta activity was normalized by dividing theta power by the sum of frequencies within 0.75 and 20 Hz over the same region. The time course of cortical excitability (that is, TEP amplitude and slope) was examined with mixed-model analyses of variance for repeated measures (PROC MIXED), with within-subject factor `circadian phase'. Contrasts were assessed with Difference of Least Square Means statement. TEP amplitude and slope were realigned, at the individual level, to dim-light melatonin onset (DLMO).

Estimation of circadian phase (where 0°=individual DLMO) was determined based on raw values. The four first samples were disregarded and maximum secretion level was set as the median of the three highest concentrations during the constant routine. Baseline level was set to be the median of the values collected from wake-up time+5 h to wake-up time+10 h. DLMOn was computed as time at which melatonin level reach 20% of the baseline to maximum difference (following linear interpolation).

ANCOVA were performed to estimate how TEP amplitude and slope were associated to theta EEG activity, subjective sleepiness and effects, cortisol level, and TMS vigilance task behavioural responses. To investigate the influence of sleep homoeostasis and circadian rhythmicity on cortical excitability, TEP amplitude and slope were fitted with, respectively, linear and sine-wave functions:

Linear function: Var=(*C*+*L* × time), where *C* corresponds to initial constant and *L* is the linear increment across time^27^.

Sine-wave function: Var=Mesor+Amplitude × sin ((sample × ti-time)/24.2), where mesor, amplitude, and time are free parameters, ti represents clock time *i* at which a sample is collected^25^.

Estimated fitted cortisol secretion profile was obtained using this same sine-wave function. The amplitude of cortisol estimated secretion, as a proxy of the circadian signal strength, was derived from the difference between the maximal and minimal cortisol predicted values.

An exponential decay function (PROC NLIN, SAS 9.3) was fitted to sleep delta data power (0.75–4 Hz) of the first four sleep cycles^70^ and derived from the frontal derivations, known to be more sensitive to sleep deprivation: SWA(*t*) =SWA0 × exp(−r × epi)^3^,^70^. The amount of initial slow wave activity (SWA0) and its dissipation rate (*r*) were derived.

Regression (PROC REG) were also performed between individual estimated cortisol amplitude and the TEP amplitude and slope decrease from session 2 to session 3 (two participants were excluded from this latter analysis because one showed a cortisol amplitude more than four standard deviations below the sample mean and another because the TMS responses of session 2 were of poor quality); 2) between individual estimated slow wave activity dissipation rate (*r*) and the TEP amplitude and slope increase from the first to the last session (four participants were excluded from this latter analysis because two showed dissipation more than three standard deviations above the sample mean and two had a TMS response during first or last session of poor quality).

### Data availability

The authors declare that the data that support the findings of this study are available from the corresponding author upon request.

## Additional information

**How to cite this article:** Ly, J. Q. M. *et al*. Circadian regulation of human cortical excitability. *Nat. Commun.* 7:11828 doi: 10.1038/ncomms11828 (2016).

## Supplementary Material

Supplementary Information Supplementary Figures 1 - 4, Supplementary Tables 1 - 2 and Supplementary References
