# Supplementary material for: Circadian regulation of human cortical excitability
Source: Nat Commun. 2016 Jun 24;7:11828. doi: 10.1038/ncomms11828 (PMC4931032; doi:10.1038/ncomms11828)
Supplement: Supplementary Information — Supplementary Figures 1 - 4, Supplementary Tables 1 - 2 and Supplementary References [file ncomms11828-s1.pdf]

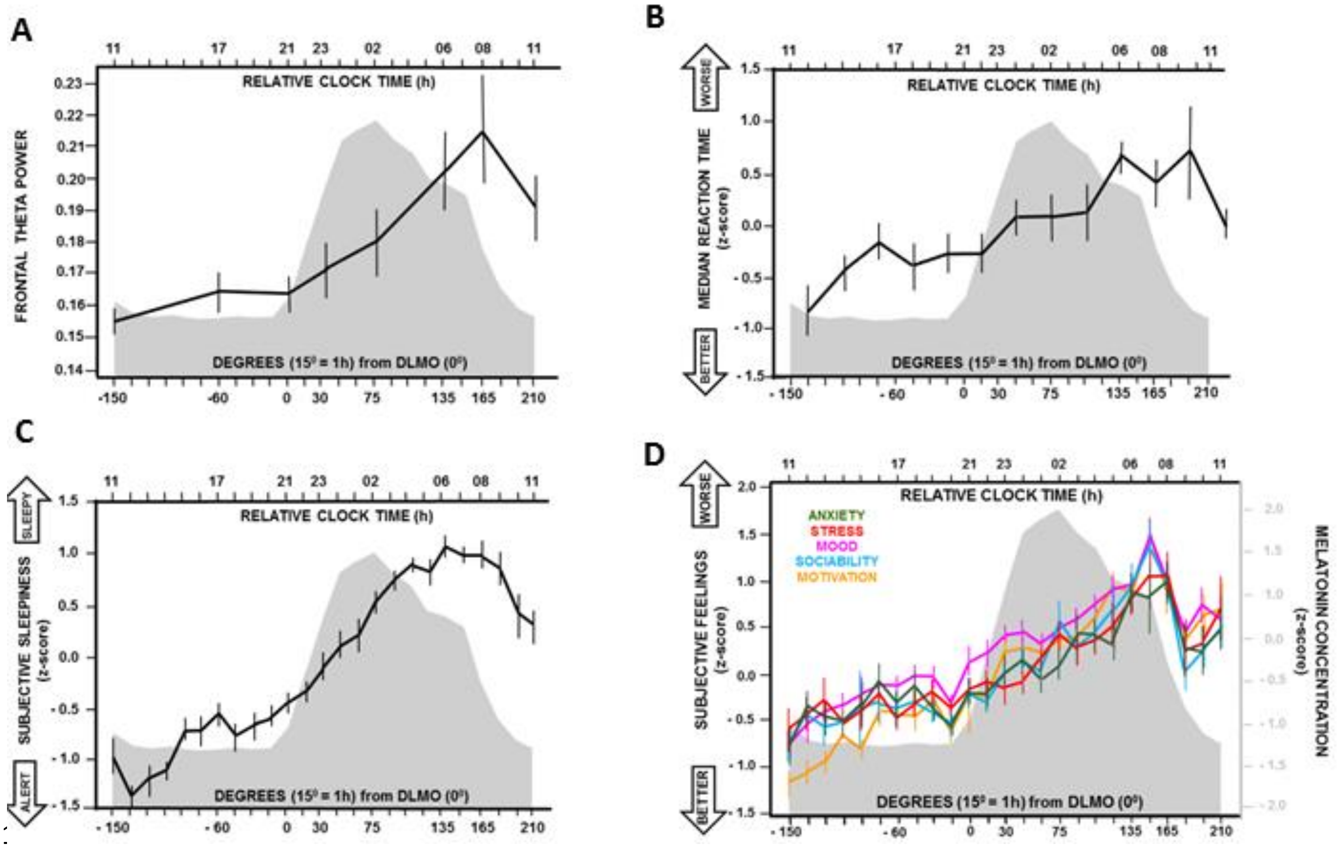

Supplementary figure 1. Time course (mean ± SD) of waking EEG frontal theta activity (A), median reaction time to the psychomotor vigilance task (B), subjective sleepiness (C) and subjective affects (D) across the protocol**.** Mean z-scored melatonin profile is displayed in background light grey. The top x axis indicates the relative clock time for a participant with an 11 pm-7am sleep wake schedule. All variables underwent typical significant variations during the protocol [PROC MIXED; n= 22 ; Theta: $F_{(7,103)}$ =3.73, p= .0012; PVT: $F_{(11,209)}$ =3.78, p< .0001; sleepiness : $F_{(24,366)}$ =17.72, p< .0001; subjective affects: $F_{(24,368)}$>4, p< .0001] with relatively stable values during the normal waking day period followed by a decrement during the biological night and subsequent partial recuperation (verified through multiple post-hoc analyses – not shown). All data are realigned to melatonin onset secretion. Due to slight delays in PVT sessions between participants, PVT was performed, as represented here, at 13 different circadian phases although there were only 12 PVT sessions per participant.

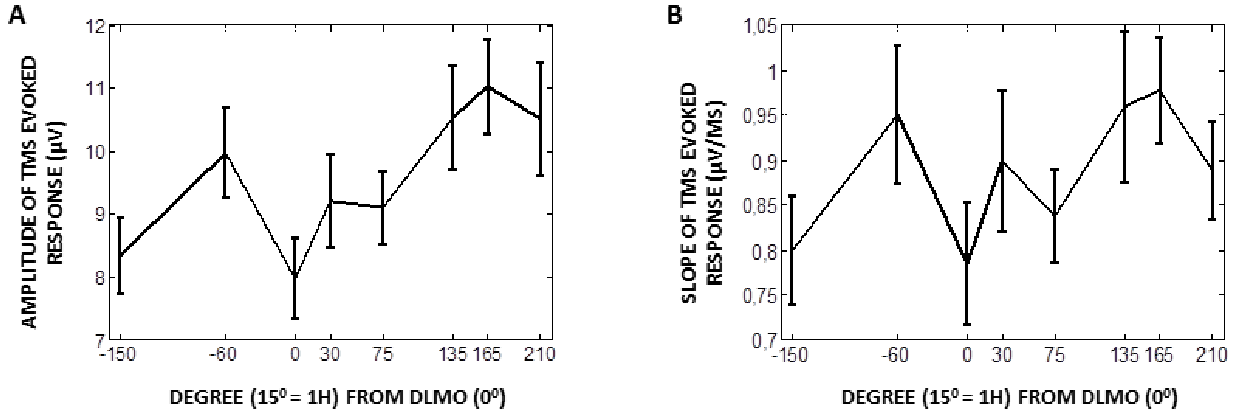

Supplementary figure 2: Time course of raw (not z-scored) measures of cortical excitability across 29h of sustained wakefulness (mean ± sem).

A. Amplitude of the TMS evoked EEG responses

B. Slope of the TMS evoked EEG responses

Raw data are displayed for completeness. Given their high skeweddness level, statistical analyses were only performed on z-scored values (see main text and Figure 2).

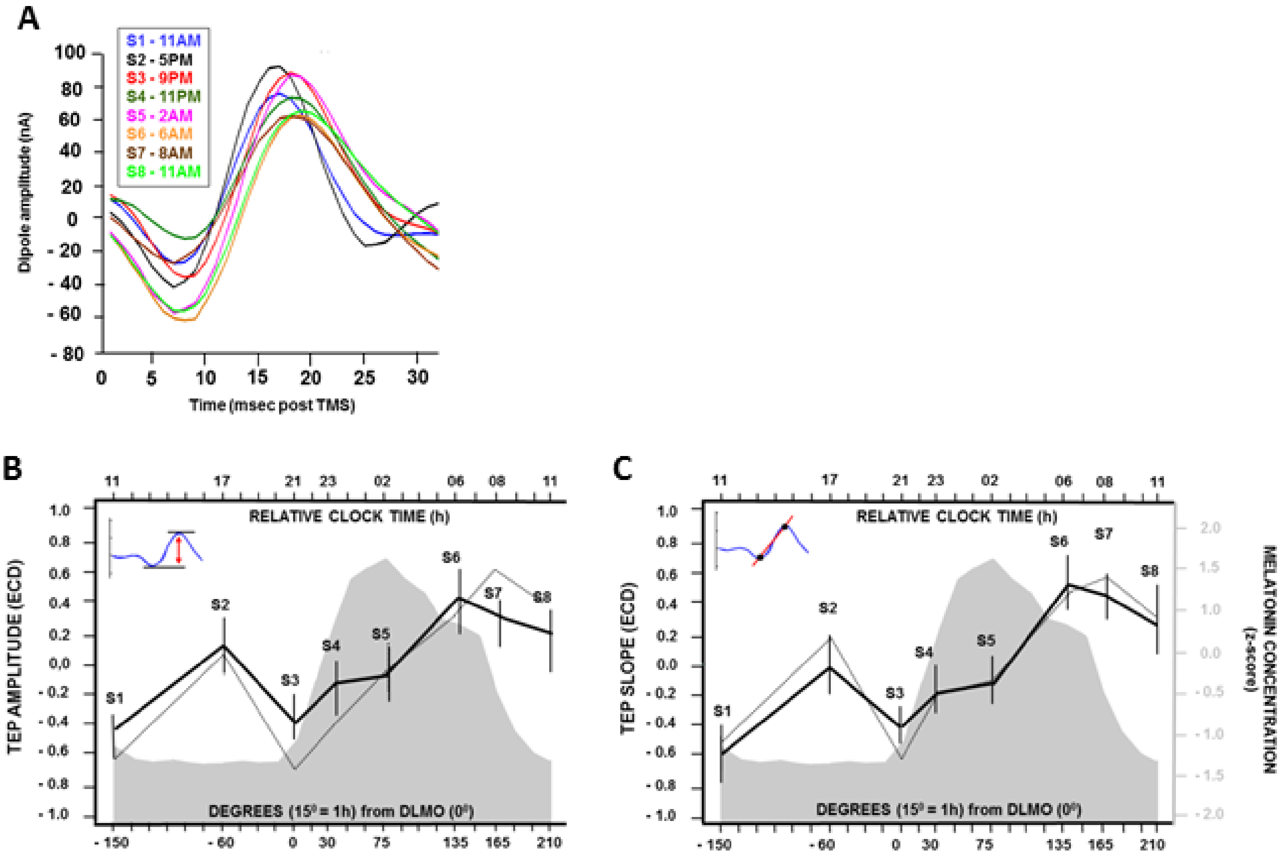

Supplementary figure 3: Cortical excitability dynamics inferred following EEG source reconstruction.

A. TMS-evoked potential (TEP) responses (0-30 ms post-TMS) computed at the hotspot after equivalent current dipole (ECD) source reconstruction, i.e. based on information for all available EEG electrodes, in a representative participant with habitual bed and wake-up times at 11 pm and 7 am, respectively.

B-C. Time course of TMS evoked response amplitude (B) and slope (C) at the hotspot based on dipole amplitude and orientation. Mean z-scored melatonin profile is displayed background in light grey. The top x axis indicates the relative clock time for a participant with an 11 pm-7am sleep wake schedule. Both indices showed significant variation with time [PROC MIXED; n=23; time effect: amplitude $F_{(7,137)}$ = 2.96, p<.0001 ; slope : $F_{(7,/137)}$ = 4.66, p <.0001]. Dashed line: TEP z-scored amplitude (cf. Fig. 2).

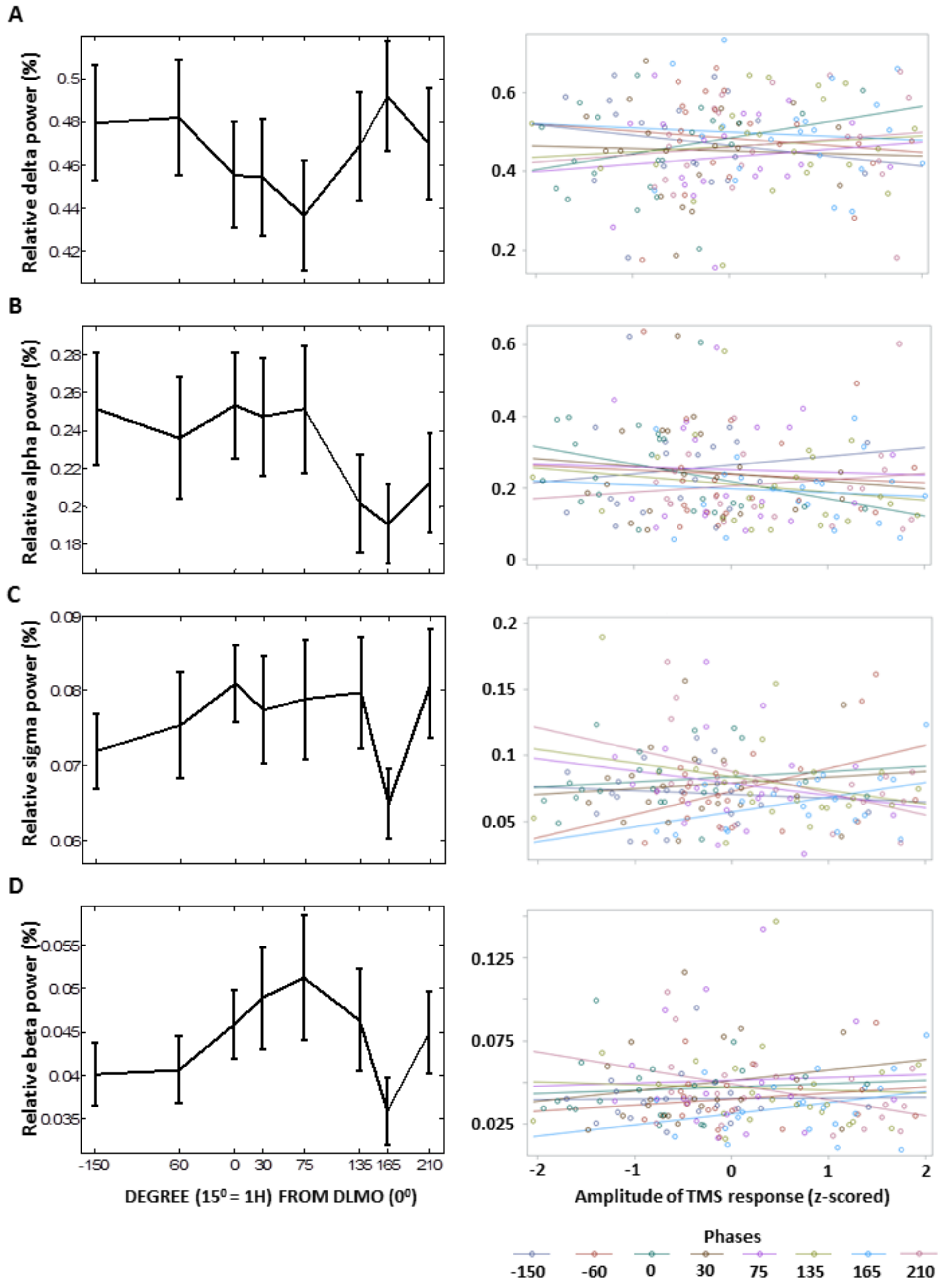

Supplementary figure 4. Changes in frontal EEG power across 29h of sustained wakefulness.

A. Relative delta power (0.75-4Hz) did not change significantly across the protocol (PROC MIXED; N = 22; main effect of circadian phase : $F_{7,109}$= 1.53, p = 0.17) and ANCOVAs (right panel)

did not reveal significant association with cortical excitability measures [AMPLITUDE (displayed): r^2= 0.04; p=0.96 – SLOPE: r^2= 0.05; p=0.95].

B. Relative alpha power (8-12Hz) changed significantly across the protocol (PROC MIXED; N = 22; main effect of circadian phase: **$F_{7,109}$= 3.79, p = 0.001**) but ANCOVAs (right panel) did not reveal significant association with cortical excitability measures [AMPLITUDE (displayed): r^2= 0.06; p=0.87 – SLOPE: r^2= 0.07; p=0.77].

C. Relative sigma power (12.5-18Hz) did not change significantly across the protocol (PROC MIXED; N = 22; main effect of circadian phase: $F_{7,111}$= 1.73, p = 0.11) and ANCOVAs (right panels) did not reveal significant association with cortical excitability measures [AMPLITUDE (displayed): r^2= 0.13; p=0.13 – SLOPE: r^2= 0.11; p=0.27].

D. Relative beta power (18.5-30Hz) did not change significantly across the protocol (PROC MIXED; N = 22; main effect of circadian phase: $F_{7,112}$= 1.84, p = 0.09) and ANCOVAS (right panels) did not reveal significant association with cortical excitability measures [AMPLITUDE (displayed): r^2= 0.08; p=0.66 – SLOPE: r^2= 0.08; p=0.67].

Supplementary Tables

Supplementary Table 1. Sample demographics (Mean ± Standard deviation), and sleep-wake timing during 7 days preceding the laboratory experiment based on sleep diary and actigraphy data (Median ± Standard deviation).

**N**	22		
**AGE**	22.82	±	2.61
**ETHNICITY**	Caucasians		
**BODY MASS INDEX**	22.23	±	2.05
**ANXIETY LEVEL (BDII)**	1.23	±	1.93
**MOOD (BECK)**	1.68	±	2.12
**DAYTIME PROPENSITY TO FALL ASLEEP (ESS)**	3.73	±	2.73
**CHRONOTYPE (HO)**	52.41	±	5.03
**RIGHT HANDED**	17/22		
**SLEEP QUALITY (PSQI)**	4.09	±	0.15
**SEASONALITY (SPAQ)**	0.64	±	0.79
**CAFFEINE (cup/day)**	0.41	±	0.50
**ALCOHOL (unit/week)**	3.41	±	0.20
**CHRONOTYPE (MCTQ)**	4.76	±	0.16
**SLEEP TIME (Sleep diary)**	23:25	±	0:20
**WAKE TIME (Sleep diary)**	7:30	±	0:17
**SLEEP DURATION (Sleep diary)**	8:10	±	0:15
**SLEEP TIME (Actigraphy)**	23:30	±	0:15
**WAKE TIME (Actigraphy)**	7:30	±	0:20
**SLEEP DURATION (Actigraphy)**	8:00	±	0:20

ANXIETY LEVEL was measured on the 21 item Beck Anxiety Inventory (BAI≤14) [1]; CHRONOTYPE was assessed by the Horne-Ösberg Questionnaire [2]; Daytime propensity to fall asleep in non-stimulating situations was assessed by the Epworth Sleepiness Scale (ESS≤11) [3]; MOOD was assessed using the 21-item Beck Depression Inventory II (BDI-II≤14) [4]; SLEEP QUALITY was determined by the Pittsburgh Sleep Quality Index Questionnaire (PSQI≤7) [5]. SEASONALITY is based on the Seasonal Pattern Assessment Questionnaire (SPAQ<11) [6]. The Edinburgh Inventory [7] was administered to verify that the participants were right-handed. Sleep parameters are presented in hours.

Supplementary Table 2. Characteristics of the 8h baseline sleep immediately preceding the sleep deprivation protocol (n = 22 ; mean ± Standard Error of the Mean).

**Baseline night Sleep Structure**			
**Total Sleep Time (h)**	7.4	±	0.15
**Sleep Efficiency (%)**	92.5	±	1.9
**Wake (min)**	27	±	4.3
**NREM stage 1 (min)**	63	±	4.5
**NREM stage 2 (min)**	220	±	7.9
**NREM stage 3 (min)**	78	±	6.2
**REM (min)**	85	±	4.1

SUPPLEMENTARY REFERENCES

1. Beck, A. T., Epstein, N., Brown, G. & Steer, R. A. An inventory for measuring clinical anxiety: psychometric properties. *J Consult Clin Psychol* **56,** 893–897 (1988).
2. Horne, J. A. & Ostberg, O. A self-assessment questionnaire to determine morningness-eveningness in human circadian rhythms. *Int J Chronobiol* **4,** 97–110 (1976).
3. Johns, M. W. A new method for measuring daytime sleepiness: the Epworth sleepiness scale. *Sleep* **14,** 540–545 (1991).
4. Steer, R. A., Ball, R., Ranieri, W. F. & Beck, A. T. Further evidence for the construct validity of the Beck depression Inventory-II with psychiatric outpatients. *Psychol Rep* **80,** 443–446 (1997).
5. Buysse, D. J., Reynolds 3rd, C. F., Monk, T. H., Berman, S. R. & Kupfer, D. J. The Pittsburgh Sleep Quality Index: a new instrument for psychiatric practice and research. *Psychiatry Res* **28,** 193–213 (1989).
6. Rosenthal N, Bradt G, W. T. Seasonal Pattern Assessment Questionaire (SPAQ). *Natl. Inst. Ment. Heal. Bethesda, MD* (1984).
7. Oldfield, R. C. The assessment and analysis of handedness: the Edinburgh inventory. *Neuropsychologia* **9,** 97–113 (1971).
